# Potential role of gut microbes in the efficacy and toxicity of immune checkpoints inhibitors

**DOI:** 10.3389/fphar.2023.1170591

**Published:** 2023-06-21

**Authors:** Jingxin Ma, Qi Wei, Xin Cheng, Jie Zhang, Zhongtao Zhang, Jianrong Su

**Affiliations:** ^1^ Department of Clinical Laboratory, Beijing Friendship Hospital, Capital Medical University, Beijing, China; ^2^ Department of General Surgery, Beijing Friendship Hospital, Capital Medical University, National Clinical Research Center of Digestive Diseases, Beijing, China; ^3^ Department of Radiology, Beijing Friendship Hospital, Capital Medical University, Beijing, China

**Keywords:** immune checkpoint inhibitors, gut microbes, efficacy, toxicity, faecal microbiota transplantation, immune-related adverse events

## Abstract

In recent years, Immune checkpoint inhibitors have been extensively used in the treatment of a variety of cancers. However, the response rates ranging from 13% to 69% depending on the tumor type and the emergence of immune-related adverse events have posed significant challenges for clinical treatment. As a key environmental factor, gut microbes have a variety of important physiological functions such as regulating intestinal nutrient metabolism, promoting intestinal mucosal renewal, and maintaining intestinal mucosal immune activity. A growing number of studies have revealed that gut microbes further influence the anticancer effects of tumor patients through modulation of the efficacy and toxicity of immune checkpoint inhibitors. Currently, faecal microbiota transplantation (FMT) have been developed relatively mature and suggested as an important regulator in order to enhance the efficacy of treatment. This review is dedicated to exploring the impact of differences in flora composition on the efficacy and toxicity of immune checkpoint inhibitors as well as to summarizing the current progress of FMT.

## 1 Introduction

The human intestine is populated by trillions of microbes (Bruneau et al., 2018; Wong and Yu, 2019), approximately 150–400 microbial species. It is typical that most of these species in the microbial community belong to the Bacteroidetes, Firmicutes, Actinobacteria, and Proteobacteria phyla (Davenport et al., 2017). As an essential part of the mammalian gut ecology, they play a key role in the maintenance of intestinal barrier homeostasis, the synthesis and metabolism of substances, and the immune surveillance of cancer (Yi et al., 2018; Peng et al., 2020), which is why gut microbes are also known as a “hidden organ” in humans. Roles of intestinal microbiota are diverse and may exchange upon completely different clinical backgrounds and host states. They can maintain the integrity of the intestinal barrier and enhance the immune response during immunotherapy. Nonetheless, they can also favor the proliferation of cancer cells, promote the growth and expansion of tumors and weaken the anti-tumor effect. Therefore, the dynamic identification of intestinal microbiota is of great importance for cancer immunotherapy (Chaput et al., 2017; Derosa et al., 2020).

Since the Food and drug administration (FDA) approval of the cytotoxic T-lymphocyte antigen-4 (CTLA-4) inhibitor ipilimumab provides effective treatment against metastatic melanoma in 2011 (Yi et al., 2018), a large number of drugs have entered into clinical trials and been in use. Compared with traditional tumor treatment methods (such as surgery, radiotherapy and chemotherapy), immune checkpoint inhibitors (ICIs) can significantly improve overall survival, reduce the rate of recurrence and delay the progression of tumors in patients with a variety of cancers (Zhang J. et al., 2022), which has brought unprecedented efficiency to advanced melanoma (Gopalakrishnan et al., 2018; Coutzac et al., 2020), renal cell carcinoma (Motzer et al., 2018; Tucker and Rini, 2020), non-small cell lung cancer (NSCLC) (He D. et al., 2021; Boesch et al., 2021) and other types of cancer. Currently, cancer immunotherapy has progressed rapidly and has become an important scientific breakthrough of cancer treatment, especially the application of ICIs like anti-programmed cell death protein 1/anti-programmed cell death 1 ligand 1 (anti-PD-1/PD-L1) and anti-CTLA-4. While early indications offer great hope for improving outcomes for cancer patients, ICIs are not without their limitations. What heads the list is that the response rates are quite low varying from 13% to 69% depending on the treatment regimen and cancer type (Topalian et al., 2012; Borghaei et al., 2015; Luke et al., 2017; Park et al., 2023), thus not all patients can benefit from the treatment. Moreover, complex and unpredictable immune-related adverse events (irAEs) may occur (Wang D. Y. et al., 2018), which refers a spectrum of unusual immunotherapy-related, potentially harmful, immunological reactions due to the generalized immune system over-reactivity and immune-mediated toxicities upon the use of the intravenous infusion of MAbs. Patients often experienced severe dermatitis, nephritis, hepatitis, arthritis, and other severe diseases (Stanley et al., 2016; Yahfoufi et al., 2023) Roughly one-third of recipients experienced these reactions during treatment and have no choice but to stop immunotherapy (Dubin et al., 2016; Anderson et al., 2019; Zhang J. et al., 2022; Thompson et al., 2022). Nowadays, mounting evidence shows that irAEs are similarly associated with the intestinal microbiota. Patients who developed ICI-related colitis have a relatively high abundance of Faecalibacterium and other Firmicutes while those without colitis have a high abundance of Bacteroidetes (Chaput et al., 2017). It may be possible to predict the risk of irAEs based on the intestinal microbiota composition.

How to modulate the microbiota to enhance the efficacy of ICIs and reduce the incidence of irAEs has become a hot topic of current research. Nowadays, flora transplantation in the form of capsules or fecal microbiota suspension is a more mature approach (Zhang J. et al., 2022), which can improve the stability of intestinal microbes and increase the abundance of intestinal flora to bring better prognosis for patients (Tan et al., 2022). Previously, the remarkable success of early trials treating *Clostrium difficile* infection by reconstitution of the gut microbiome is cause for measured but realistic hope (McKenney and Pamer, 2015; Smd et al., 2020). Subsequently, fecal microbiota transplantation was successfully promote response in a small number of ICIs refractory melanoma patients (Baruch et al., 2021). Therefore, this review aims to clarify the relationships between microorganisms and the efficacy and irAEs of ICIs. Additionally, we are dedicated to pointing out opinions on how to modulate microorganisms to enhance the quality of life for patients with advanced malignant tumors and reduce treatment side effects.

## 2 Gut microbiome modulates the efficacy of immunotherapy

### 2.1 Gut microbiome modulation of ICIs treatment efficacy in different types of solid tumors

In recent years, several studies have demonstrated that the composition of intestinal microbiome is associated with the efficacy of immunotherapy. Through quantitative metagenomics using next-generation sequencing, quantitative polymerase chain reaction or 16S ribosomal RNA sequencing, the researchers were able to analyze the composition of the intestinal microbiota as well as functions of microbiota which are beneficial to identify the responders who experienced immunotherapy. 16S ribosomal RNA sequencing has provided a more complete picture of the compositon of microbial inhabitants of the gut (Lamendella et al., 2012; Zhang H. et al., 2022), which based on the variable regions (V3-V4) (Whon et al., 2021). Nonetheless, the information on the functional relationships within microbial communities, or between the microbiota and the human host is very limited. Therefore, The more costly metagenomic next-generation sequencing could help identify bacteria on species level and obtain potential functional insight although a wealth of functions unknown (Hajjo et al., 2022; Zwezerijnen-Jiwa et al., 2023). To explore and understand microbial phylogenetic and functional compositions in human gut microbiota, nucleic acid sequencing can be offered. These approaches have enabled the characterization of the phylogenetic and functional microbial communities inhabiting the gut, which will be important for future diagnostic instruments for various diseases (Cong and Zhang, 2018). Nowadays, reports on the relationship between the gut microbiota and immune efficacy mainly focus on seven types of cancer, as shown in Table 1. Metastatic melanoma (MM) and non-small cell lung cancer (NSCLC) account for the highest proportion among them. These intricate interplays will be elaborated in detail following.

**TABLE 1:** Studies about the relationship between the gut microbiome and response to immune checkpoint inhibitor

cancer site	author	year	ICI	sample type	accessment method	patients	R(n)	NR(n)
MM	Brandilyn A. Peters et al.	2019	Anti-PD1 Anti-CTLA4	fecal	16SrRNA+mNGS	27	**Firmicutes:** *Faecalibacterium* **Bacteroidetes:** *Parabacteroides*	**Proteobacteria:** *Bilophila* **Bacteroidetes:** *Bacteroides ovatus* **Firmicutes:** *Blautia producta, Ruminococcus gnavus*
MM	Matson et al.	2018	Anti-PD1 Ipilimumab	fecal	16SrRNA+mNGS	42	**Firmicutes:** *Enterococcus faecium, Veillonella parvula, Lactobacillus* **Actinobacteria:** *Collinsella aerofaciens, Bifidobacterium adolescentis, Bifidobacterium longum* **Proteobacteria:** *Klebsiella pneumoniae* **Bacteroidetes:** *Parabacteroides merdae*	**Firmicutes:** *Ruminococcus obeum, Roseburia intestinalis*
MM	N. Chaput et al.	2017	Ipilimumab	fecal	16SrRNA	26	**Firmicutes:** *Ruminococcus, Lachnospiraceae, Faecalibacterium*	**Bacteroidetes:** *Bacteroides*
MM	Miles Andrews et al.	2021	ipilimumab either nivolumab or pembrolizumab	fecal	16SrRNA+mNGS	77	**Bacteroidetes:** *Bacteroides stercoris, Parabacteroides distasonis* **Firmicutes:** *Fournierella massiliensis*	**Proteobacteria:** *Klebsiella aerogenes* **Firmicutes:** *Lactobacillus rogosae*
MM	Frankel et al.	2017	Ipilimumab nivolumab Pembrolizumab	fecal	mNGS	39	**Firmicutes:** *Streptococcus parasanguinis, Dorea formicigenerans* **Bacteroidetes:** *Bacteroides caccae*	**Firmicutes**: *Faecalibacterium prausnitzii, Holdemania filiformis* **Bacteroidetes:** *Bacteroides thetaiotaomicron*
MM	Rebecca C. Simpson et al.	2022	nivolumab ipilimumab	fecal	16SrRNA	103	**Firmicutes:** *Faecalibacterium prausnitzii, Butyricicoccus pullicaecorum* **Verrucomicrobia:** *Akkermansia muciniphilia*	**Bacteroidetes:** *Bacteroidaceae*
MM	V.Gopalakrishnan et al.	2018	PD1	fecal	16SrRNA+mNGS	112	**Firmicutes**: **16s:** *Clostridiales, Ruminococcaceae* **mNGS:** *Faecalibacterium*	**Bacteroidetes: 16s:** *Bacteroidales* **mNGS**: *Bacteroides thetaiotaomicron* **Proteobacteria:** *Escherichia coli* **Firmicutes:** *Anaerotruncus colihominis*
MM	Diwakar Davar et al.	2021	pembrolizumab	fecal	mNGS	15	**Firmicutes:** *Lachnospiraceae, Ruminococcaceae* **Actinobacteria:** *Bifidobacteriaceae, Coriobacteriaceae*	**Bacteroidetes**
NSCLC	Peng Song et al.	2020	Anti-PD1	fecal	mNGS	63	**Bacteroidetes:** *Parabacteroides* **Euryarchaeota:** *Methanobrevibacter*	**Firmicutes:** *Veillonella, Selenomonadales, Negativicutes*
NSCLC	Jin et al.	2019	Nivolumab	fecal	16SrRNA	25	**Bacteroidetes:** *Alistipes putredinis, Prevotella copri* **Actinobacteria:** *Bifidobacterium longum* **Firmicutes:** *Lachnobacterium, Lachnospiraceae* **Proteobacteria:** *Shigella*	**Firmicutes:** *Ruminococcus* **Actinobacteria:** *Bifidobacterium longum* **Bacteroidetes:** *Prevotella copri*
NSCLC	Yueping Jin et al.	2019	nivolumab	fecal	16SrRNA	77	**Bacteroidetes:** *Alistipes putredinis, Prevotella copri* **Actinobacteria:** *Bifidobacterium longum*	**Firmicutes:** *Ruminococcus*
NSCLC	Chao Fang et al.	2022	nivolumab camrelizumabpembrolizumab	fecal	mNGS	85	**Bacteroidetes:** *Bacteroidesmassiliensis, prevotellaceae, Alistipes obesi*	**Firmicutes:** *Enterocloster* **Bacteroidetes:** *Bacteroides fragilis*
NSCLC	Taiki Hakozaki et al.	2021	nivolumab, pembrolizumab, or atezolizumab	fecal	16SrRNA	70	**Firmicutes:** *Ruminococcaceae UCG 13, Agathobacter,Lachnospiraceae UCG001*	NA
NSCLC	Rachel C. Newsome et al.	2022	Anti-PD1 Anti-CTLA4	fecal	16SrRNA	65	**Firmicutes:** *Ruminococcus, Faecalibacterium* **Verrucomicrobia:** *Akkermansia*	NA
NSCLC,RCC	Routy et al.	2018	Anti-PD1	fecal	mNGS	78	**Verrucomicrobia:** *Akkermansia muciniphila* **Firmicutes:** *Ruminococcus,Eubacterium* **Bacteroidetes:** *Alistipes*	NA
Thoracic-carcinoma	Huihui Yin et al.	2021	Anti-PD1	fecal	16SrRNA	42	**Verrucomicrobia:** *Akkermansiaceae* **Firmicutes:** *Enterococcaceae, Carnobacteriaceae, Clostridiales Family XI bacterial families* **Proteobacteria:** *Enterobacteriaceae*	NA
RCC	Lisa Derosa et al.	2020	nivolumab	fecal	mNGS	58	**Bacteroidetes:** *Alistipes senegalensis, Bacteroides salyersiae* **Firmicutes:** *Clostridium ramosum* **Verrucomicrobia:** *Akkermansia muciniphila*	**Firmicutes:** *C. hathewayi, Clostridium clostridioforme*
HCC	Jinzhu Mao et al.	2021	Anti-PD1	fecal	mNGS	65	**Bacteroidetes**: *Alistipes sp Marseille-P5997* **Firmicutes:** *Ruminococcus calidus, Erysipelotichaceae bacterium-GAM147, Lachnospiraceae bacterium-GAM79*	**Firmicutes:** *Veillonellaceae*
HCC	Lili LI et al.	2020	Anti–PD-1	Buccal+fecal	16SrRNA	65	**Firmicutes:** *Clostridiales, Ruminococcaceae*	**Bacteroidetes:** *Bacteroidales*
HCC	Zheng et al.	2019	camrelizumab	fecal	mNGS	8	**Firmicutes:** *four Lactobacillus species (L. oris, L. mucosae, L.gasseri, and L. vaginalis), Streptococcus thermophilus* **Actinobacteria:** *Bifidobacterium dentium*	NA
GICA	Peng et al.	2020	Anti–PD-1 CTLA-4 blockade	fecal	16SrRNA+mNGS	74	**Verrucomicrobia:** *Akkermansia* **Bacteroidetes:** *Prevotellaceae, Prevotella/Bacteroides, Parabacteroids* **Firmicutes:** *Lachnoclostridium, Lachnospiraceae, Ruminococcaceae, Flavonifractor(Eubacterium),Dialister*	**Bacteroidetes:** *Bacteroides, Parabacteroides* **Firmicutes:** *Coprococcus, Subdoligranulum*
ESCC	Liwei Xu et al.	2022	camrelizumab	fecal	16SrRNA	46	**Bacteroidetes:** *Barnesiellaceae, Odoribacteraceae, Butyricimonas, Prevotella, Barnesiella, Odoribacter* **Synergistetes:** *Dethiosulfovibrionaceae, Pyramidobacter genus*	**Proteobacteria:** *Aeromonadales, Pseudomonadales, Moraxellaceae, Rhodocyclales, Rhodocyclaceae, Acinetobacter* **Fimicutes:** *Dialister* **Deinococcus-Thermus:** *Deinococci*
Pan-carcinoma	Zhaozhen Wu et al.	2022	Anti-PD1	fecal	mNGS	27	**Bacteroidetes:** *Parabacteroides* **Firmicutes:** *Clostridia bacterium UC5.1_2F7* **Actinobacteria:** *Bifidobacterium dentium*	**Bacteroidetes:** *Bacteroides dorei* **Actinobacteria:** *Nocardia*

#### 2.1.1 Bacterial markers for immunotherapy against metastatic melanoma

Several studies on patients with metastatic melanoma revealed that there was a significant difference in the diversity of intestinal microbiome between those who responded to anti-PD-1 treatment and those who did not. In metastatic melanoma, Firmicutes were found to be more frequent in responders. Additionally, the diversity of Bacteroidetes was notably higher among those who did not respond (Frankel et al., 2017; Gopalakrishnan et al., 2018a; Matson et al., 2018; Jin et al., 2019; Martin et al., 2019; Peters et al., 2019; Derosa et al., 2020; Peng et al., 2020; Song et al., 2020; Andrews et al., 2021; Mao et al., 2021; Fang et al., 2022; Xu et al., 2022). The Proteobacteria phylum was more commonly found in the intestinal flora of non-responders to metastatic melanoma. However, Matson et al. discovered an enrichment of *Klebsiella pneumoniae* (belonging to Proteobacteria phylum) in the feces of patients who responded to programmed cell death protein 1(PD1) treatment. Actinobacteria and Verrucomicrobia phylum were the only ones present in the intestinal flora of metastatic melanoma patients who responded to immunotherapy (Matson et al., 2018; Davar et al., 2021; Simpson et al., 2022), suggesting that these may be the dominant bacteria in responders. It is unclear, however, how the specific dominant phyla in metastatic melanoma may influence tumor immune effects in patients.

The appearance of paradox may be associated with microbiota-derived metabolites, such as those produced by *Clostridales* in the Fimicutes phylum and *Akkermansia municiphilla* in the Verrucomicrobia phylum (Louis et al., 2014; Morrison and Preston, 2016; Martin-Gallausiaux et al., 2021). These metabolites may enhance or diminish antitumor efficacy through immunoregulation. Favorable metabolites include short chain fatty acids, polysaccharide A, inosine, polyamines, long chain fatty acids, tryptophan derivatives and trimethylamine N-oxide. For example, Short chain fatty acids (SCFAs) are products of fiber fermentation by intestinal bacteria, which contain acetic acid, propionic acid, butyric acid, valerate and so on. SCFAs can provide energy for the colon cells and inhibit various cancer signaling pathways and inflammatory responses (Donohoe et al., 2011; Chen et al., 2019), such as the NF-κB and its downstream pathways to reduce the release of inflammatory factors (Trompette et al., 2018; He Y. et al., 2021; Zhang J. et al., 2022). Among them, Butyric acid produced by *prausnitzii* can promote the proliferation of CD8^+^T and enhance anti-tumor immunity (Bachem et al., 2019). Mucin synthesis can be induced and intestinal mucosal integrity can be maintained on the basis of SCFAs (Guo and Li, 2019). In addition, SCFAs can stimulate DNA mismatch repair genes to increase the ability of gene expression and promote gene stability, which can also induce differentiation and apoptosis of colorectal cancer cells (Sun and Zhu, 2018). Thus, SCFAs have the potential to be used as biomarkers for the efficacy of immunotherapy (Nomura et al., 2020). Another study found Polysaccharide A (PSA), which is secreted by *Bacteroides fragilis* in the colon, can activate CD4^+^T and promote the release of IL-10 to suppress inflammation (Wang et al., 2006; Round et al., 2011). The metabolites of *Bifidobacterium pseudobifidum* and *A. muciniphila*——inosine can bind to A2A receptors on the surface of T cells to enhance antitumor immunity and enhance the efficacy of ICIs (Mager et al., 2020). It happens that there is a similar case that Hai Wang et al. found that the trimethylamine N-oxide produced by *Clostridiales* can enhance the efficacy of immunotherapy in triple-negative breast cancer, which is proportional to CD8^+^T cell (Wang et al., 2022). While adverse metabolites contain N-nitroso compounds, bile acids, ammonia, phenols, hydrogen sulfide, lipopolysaccharide and so on. Lipopolysaccharide is the metabolite of Gram-negative bacterial, which can promote immune escape in CRC cells through the activation of TLR4 and the induction of immunosuppressive factors (Li et al., 2014). Ammonia, phenols, and hydrogen sulfide create chronic inflammation and induce DNA damage leading to CRC development, the same as N-nitroso compounds (Ijssennagger et al., 2016; Borzì et al., 2018; Mizutani et al., 2020). Consequently, we can conclude that metabolic approach can suggest potentials in personalized management through helping prediction of efficacy process of immunotherapy.

#### 2.1.2 Effects of the gut microbiota on non-small cell lung carcinoma

In patients with non-small cell lung carcinoma, both the Firmicutes phylum and the Bacteroidetes phylum are present in both responders and non-responders. *Bifidobacterium longum* in the Actinobacteria phylum and *A. muciniphila* in the Verrucomicrobia are beneficial bacteria that are enriched in immune responders (Routy et al., 2018a; Jin et al., 2019). *Bifidobacterium* has immunomodulatory effects and is closely related to the energy metabolism of regulatory T cells, which may improve the symptoms of colitis through the accumulation of conjugated linoleic acid (Zhou et al., 2022). *A. muciniphila* can produce inosine, induce the expression of TH1 regulatory genes in CD4^+^ T cells (Zhang et al., 2019), and reverse PD-1 blockade by IL-12 from dendritic cells, increasing the recruitment of CCR9^+^ CXCR3^+^ CD4^+^ T lymphocytes to the tumor microenvironment to kill tumor cells (Routy et al., 2018b). It has been found to be abundant in NSCLC, MM, GI tumors, and renal cell cancer responders, making it a potential microbial marker of response to immune checkpoint therapy^54^. Akkermansia muciniphila may also have epidemiological links to inflammation (Derosa et al., 2022), reduce obesity and its complications (Zhou et al., 2020), alleviate neurodegenerative diseases (Blacher et al., 2019) and inhibit premature aging (Bárcena et al., 2019).

#### 2.1.3 Potential role of gut microbiota on other types of cancers

In patients with hepatocellular carcinoma and renal cell carcinoma, the Firmicutes phylum was more abundant in the fecal flora of patients who responsed to immunotherapy, while the Bacteroidetes phylum was relatively abundant in the fecal flora of those who did not respond (Routy et al., 2018a; Derosa et al., 2020; Li and Ye, 2020; Mao et al., 2021). Additionally, *bifidobacteria* was only found in the feces of patients with hepatocellular carcinoma patients who responded (Zheng et al., 2019), and *A. muciniphila* was only found in the feces of renal cell carcinoma patients (Routy et al., 2018a; Derosa et al., 2020), These findings suggest that the Firmicutes, Actinobacteria, and Verrucomicrobia phyla may be indicator markers for both cancers, providing valuable insight into the efficacy and prognosis of immunotherapy. Unfortunately, the gut microbiota is dynamic and evolves with the pathology. Confounding environmental factors may influence the composition of it, such as diet, medication, smoking and other lifestyle factors (Huxley et al., 2009; Conlon and Bird, 2015). So we shall make the best of our ability to control these factors including patient demographics (sex, age, race, comorbidities) (Gong et al., 2019). Besides, the same bacteria in distinct communities can have different functions in the interation with the host, which may predict contradictory prognosis. Hence, large cohorts, and clinical trials should be performed to assess the impact of gut microbiota on the effectiveness of ICIs (Rezasoltani et al., 2021; Roviello et al., 2022).

Similarly, there is a lack of literature on the relationship between immunotherapy efficacy and intestinal flora in gastrointestinal tract tumors. Peking University Cancer Hospital studied the changes in the flora of 74 GI tract tumor patients before and after treatment with immune checkpoint inhibitors and found that the composition of the patients’ body flora and gut microbial metabolites affect the patients’ response to programmed cell death protein 1/programmed cell death 1 ligand 1(PD1/PDL1) antibodies. Specific response groups exhibited high abundance of Prevotella, Ruminococcaceae and Lachnospiraceae, all of which belong to the Firmicutes phylum. Additionally, *Eubacterium, Lactobacillus and Streptococcus* in different GI tumor types were positively correlated with the therapeutic response to PD1/PDL1 inhibitors. Furthermore, *Blue-green algae,* Lachnospiraceae*, Ruminococcus and Microbacterium* were all enriched in patients benefiting from colorectal cancer immunotherapy. This study highlights that gut microbes can predict response efficacy and can serve as potential biomarkers of response to immune checkpoint inhibitors. Liwei Xu et al. found a special phylum—Synergistetes, which were abundant in clinical responders of esophageal squamous cell carcinoma. Synergistetes is a rare class of anaerobic bacteria (McCracken and Nathalia Garcia, 2021) and have frequently been reported in the human oral cavity at sites of dental disease, especially periodontitis. Although Synergistetes are pathogenic, they favored the efficacy of immunotherapy in patients, thus more clinical studies and trials are needed to verify this. Moreover, Emerging evidence points that the alpha diversity is not necessarily a positive correlation with the immunotherapeutic efficacy. Huihui Yin et al. discovered that patients with a higher commensal bacterial abundance had a prolonged progression-free survival (PFS) (Yin et al., 2021). While another study did not observe statistically significant differences in bacterial taxa relative abundance between responders and non-responders. The interpretability of findings may originate from the variation of each study design and the data analyses (Peng et al., 2020). The Akkermansiaceae, Enterococcaceae, Carnobacteriaceae, and Clostridiales Family XI were all over-represented at diagnosis in patients with longer PFS (Yin et al., 2021). These studies highlight that gut microbes can predict response efficacy and can serve as potential biomarkers of response to immune checkpoint inhibitors. Therefore, the composition of intestinal microbiome plays a key role in cancer immunotherapy.

### 2.2 Intricate interplay between the gut microbiota and differential immunotherapeutic efficacy

Based on our statistical study, we found that Firmicutes were present in the fecal flora of responders of 19 reports across 23 studies, Bacteroidetes were present in the fecal flora of responders in 14 studies, and Actinobacteria phylum was found to have significant immune efficacy. Proteobacteria phylum, however, is controversial in its contribution to immune efficacy. *Klebsiella pneumonia*, *Shigella* and Enterobacteriaceae in Proteobacteria phylum were reported to be present in the gut microbiome of patients with responders (Matson et al., 2018; Jin et al., 2019; Yin et al., 2021). However, Liwei Xu, Brandilyn A. Peters, Miles Andrews et al. all discovered that Proteobacteria phylum was widely present in the feces of non-responding patients in their studies (Gopalakrishnan et al., 2018a; Peters et al., 2019; Xu et al., 2022). Six orders of Proteobacteria were associated with non-responders, including Aeromonodales, Pseudomonadales, Moraxellales, Rhodocyclales, Desulfovibrionales, and Enterobacterales, and were associated with shorter progression-free survival and impaired antitumor immune responses mediated by limited intratumoral lymphoid and weakened antigen presentation capacity (Gopalakrishnan et al., 2018a). The exact mechanisms by which this occurs remain unclear, and more evidence is needed to explore it. In conclusion, Verrucomicrobia, Euryarchaeota, and Synergistetes were only present in patients with responders, while Deinococcus-Thermus was present in patients without responders, as detailed in Table 2. Therefore, according to the above researches, Fimicutes, Bacteroidetes, Verrucomicrobia, Euryarchaeota, and Synergistetes phylum may be the potential biomarkers for cancer immunotherapy.

**TABLE 2:** Gut microbiome bacteria in responders and non-responders to immune checkpoint inhibitors, by phylum.

Responders	Phylum							
	Firmicutes	Bacteroidetes	Actinobacteria	Proteobacteria	Verrucomicrobia	Euryarchaeota	Synergistetes	Deinococcus-Thermus
Yes	*Agathobacter, Butyricicoccus pullicaecorum,* Carnobacteriaceae*, Clostridiales, Clostridiales Family XI bacterial families, Clostridia bacterium UC5.1_2F7, Clostridium ramosum, Dialister, Dorea formicigenerans,* Enterococcaceae*,* Erysipelotichaceae *bacterium-GAM147, Enterococcus faecium, Eubacterium, Faecalibacterium prausnitzii, Fournierella massiliensis, Flavonifractor,* Lachnospiraceae *bacterium-GAM79,* Lachnospiraceae*, Lachnobacterium,* Lachnospiraceae *UCG001, Lactobacillus, Lachnoclostridium, Ruminococcus, Ruminococcus calidus,* Ruminococcaceae*,* Ruminococcaceae *UCG 13, Streptococcus parasanguinis, Streptococcus thermophilus, Veillonellaparvula*	*Alistipes obesi, Alistipes putredinis, Alistipes senegalensis, Alistipes sp Marseille-P5997, Bacteroides caccae, Bacteroides massiliensis, Bacteroides stercoris, Bacteroides salyersiae,* Barnesiellaceae*, Barnesiella, Butyricimonas,* Odoribacteraceae*, Parabacteroides merdae, Prevotella copri,* Prevotellaceae*, Parabacteroides distasonis*	*Bifidobacterium adolescentis, Bifidobacterium longum, Bifidobacterium dentium,* Bifidobacteriaceae*,* Coriobacteriaceae*, Collinsella aerofaciens*	*Klebsiella pneumoniae, Shigella,* Enterobacteriaceae	*Akkermansia muciniphila*	*Methanobrevibacter*	Dethiosulfovibrionaceae*, Pyramidobacter*	—
No	*Anaerotruncus colihominis*	*Bacteroides, Bacteroidales, Bacteroidesthetaiotaomicron,* Bacteroidaceae*, Bacteroides ovatus, Bacteroides fragilis, Bacteroides dorei, Prevotella copri, Parabacteroides*	*Bifidobacterium longum, Nocardia*	*Aeromonadales, Acinetobacter, Bilophila, Klebsiella aerogenes,* Moraxellaceae*, Rhodocyclales, Pseudomonadales,* Rhodocyclaceae	*—*	*—*	*—*	*Deinococci*
	*Blautia producta, Coprococcus*							
	*C.hathewayi, Clostridium clostridioforme, Dialister, Enterocloster, Faecalibacterium prausnitzii, Holdemania filiformis, Lactobacillus rogosae, Negativicutes, Ruminococcus obeum, Roseburia intestinalis, Ruminococcus gnavus, Subdoligranulum, Selenomonadales, Veillonella,* Veillonellaceae							

### 2.3 Animal testing to verify the interplay between gut microbiome and host immunity

Based on the above studies, we found modulating intestinal flora can affect the efficacy of immune checkpoint inhibitors. To a certain degree, several animal studies have now demonstrated that intervention of intestinal flora can enhance the treatment of immune checkpoint inhibitors. Yoon et al. (2021) combined Bifidobacterium shortum and PD1 inhibitors in mice and found that both CD8^+^ T cell levels and CD8^+^/Treg ratios were elevated in mice, increasing the anti-tumor efficacy of mice (Yoon et al., 2021). Similarly, Montalban-Arques et al. used PD1 inhibitors along with a mixture of four *Clostridium* species instilled into the stomachs of mice and found that CD8^+^ T cells were infiltrated around the tumor tissue. As a result, this combination treatment cleared almost all tumor cells (Montalban-Arques et al., 2021) and achieved a better synergistic effect. However, all of the above are animal trials and more clinical trials are needed to explore and validate.

## 3 Gut microbiota in immune-related toxicity

Although immunotherapy has brought a revolutionary breakthrough in cancer treatment, the use of CTLA4 and PD1 blockers can lead to an over-activation of the immune system, resulting in increased intestinal permeability and loss of intestinal barrier integrity, which can cause systemic inflammation and immune-related adverse events (irAEs). Thus, the benefits associated with ICIs come at the cost of irAEs, and the increased efficacy is usually accompanied by irAEs. Unlike typical chemotherapy-related toxicity, it can be considered of off-target effects of an over-activated immune system (F et al., 2019), immune-related adverse events often manifest as immune-associated colitis (Liu Z. et al., 2021), diarrhea (Kelly-Goss et al., 2022), rash (Dimitriou et al., 2019), arthritis (Kostine et al., 2021) and so on (Stanley et al., 2016). Higher abundance of gut microbiota has been observed in patients experiencing mild diarrhea compared to those with severe diarrhea, suggesting that enrichment of the gut microbiota is important for the prevention of irAEs.

### 3.1 The gut microbiome and irAE occurrence: a new adventure world

Studies on flora and immune-related adverse events focused on five solid tumors (Table 3), in detail, patients without irAEs or with irAEs showed an abundance of 7 abundant bacteria in the phylum level (Table 4). The study found that the Firmicutes phylum was associated with a high probability of adverse events with immunotherapy, while the Bacteroidetes phylum was associated with a low probability of immune-related adverse events. Of the 9 articles studied, 6 articles found Firmicutes to be enriched in groups with immune-related adverse events, while Bacteroidetes phylum was similarly found in groups without immune-related adverse events. On the contrary, Mao et al. conducted a metagenomic analysis of stools from 65 patients with hepatocellular carcinoma with different responses and found that immune-associated colitis was largely associated with low diversity and abundance of gut microorganisms. Bacteroidetes phylum was found to cause more severe immune-related adverse events and is a potential biomarker for predicting severe diarrhea and colitis, while the high abundance and diversity of Firmicutes phylum may be a protective factor against immunotherapy-induced toxicity (Mao et al., 2021). However, the exact mechanism of this is still unknown and requires further research to be proven.

**TABLE 3:** Studies that access the composition of the gut microbiome with irAEs or without irAEs

cancer site	author	time	method	sample type	irAEs	no irAEs
MM	Krista Dubin et al.	2016	16S rRNA	fecal	*Low Bacteroidaceae*	**Bacteroidetes:** *Bacteroidaceae, Rikenellaceae, Barnesiellaceae*
MM	chaput et al.	2017	16S rRNA	fecal	**Firmicutes:** *Lachnospiraceae, Ruminococcacea* **Bacteroidetes:** *Bacteroidaceae*	**Bacteroidetes:** *Prevotellaceae Bacteroidaceae, Porphyromonadaceae* **Firmicutes:** *Ruminococcaceae*
UCC	Daniel Y. Wang et al.	2018	16S rRNA	fecal	**Proteobacteria:** *Escherichia* **Firmicutes:** *Clostridia*	NA
HCC	Jinzhu Mao et al.	2021	16S rRNA	fecal	**Bacteroidetes**	**Firmicutes**
MM	Miles Andrews et al.	2021	16S rRNA+mNGS	fecal	**Bacteroidetes:** *Bacteroides intestinalis* **Firmicutes:** *Intestinibacter bartlettii*	**Firmicutes:** *Dorea formicigenerans*
NSCLC	Taiki Hakozaki et al.	2021	16S rRNA	fecal	3-4: **Firmicutes:** *Agathobacter*1-2: **Verrucomicrobia**: *Akkermensia* **Firmicutes:** *Lactobacillaceae* **Proteobacteria:** *Raoultella*	**Firmicutes:** *Lactobacillaceae* **Proteobacteria:** *Raoultella*
Pan-carcinoma	Wenhui Liu et al.	2021	16S rRNA	fecal	3-4:**Bacteroidetes**:*Spirosomaceae* **Firmicutes:** *Thermoanaerobacteracea*,*Streptococcus* **Proteobacteria:** *Anaplasmataceae*, *Vibrionales*, *Stenotrophomonas* 1-2: **Firmicutes:** *Faecalibacterium, unidentified_ Lachnospiraceae* **Actinobacteria:** *Nocardiaceae* **Proteobacteria:** *Pseudomonadaceae*	**Bacteroidetes**:*Balneolales* **Proteobacteria:** *Pseudomonadales*
MM	Rebecca C. Simpson et al.	2022	16S rRNA	fecal	**Bacteroidetes:** *Bacteroidaceae*	**Firmicutes:** *Faecalibacterium prausnitzii, Oscillospira, Ruminococcus bromii, Lachnospiraceae*
ESCC	Liwei Xu et al.	2022	16S rRNA	fecal	≥3:**Firmicutes:** *Succiniclasticum, Staphylococcus* **Actinobacteria:** *Nakamurella, Actinosynnemataceae, Lentzea, Pseudonocardia* **Proteobacteria:** *Rhizobium, Chelativorans, Phyllobacteriaceae, Pelagibacteraceae, Coxiellaceae Acidicapsa, Plesiomonas* **Acidobacteria:** *Granulicella, Acidobacteriaceae, bacterium Ellin6075* **Bacteroidetes:** *Aquirestis, Flavisolibacter, Dysgonomonas* 1-2: **Firmicutes:** *Phascolarctobacterium, Anaerotruncus* **Bacteroidetes:** *Odoribacteraceae,Odoribacter, Butyricimonas* **Synergistetes:** *Synergistia, Synergistales, Synergistes* **Proteobacteria:** *Deltaproteobacteria*	NA

MM: metastatic melanoma; UCC: urothelial cell carcinoma; HCC: hepatocellular carcinoma; NSCLC: non-small cell lung carcinoma; ESCC: esophageal squamous cell carcinoma; NA: not assessed; 16S rRNA:16S ribosomal RNA sequencing; mNGS:metagenomic next generation sequencing

**TABLE 4:** Gut microbiome bacteria in patients with irAEs or without irAEs, by phylum.

irae	Phylum						
	Firmicutes	Bacteroidetes	Proteobacteria	Actinobacteria	Verrucomicrobia	Acidobacteria	Synergistetes
Yes	*Anaerotruncus, Agathobacter, Clostridia, Faecalibacterium, Intestinibacter bartlettii,* Lachnospiraceae*,* Lactobacillaceae*,* Ruminococcaceae*, Streptococcus, Succiniclasticum, Staphylococcus, Thermoanaerobacteracea*, *Phascolarctobacterium*	*Aquirestis,* Bacteroidaceae*, Bacteroides, Butyricimonas intestinalis, Dysgonomonas, Flavisolibacter,* Odoribacteraceae*, Odoribacter,* Spirosomaceae	Anaplasmataceae *Acidicapsa, Chelativorans,* Coxiellaceae *Plesiomonas, Deltaproteobacteri, Escherichia,* Pseudomonadaceae*,* Phyllobacteriaceae*,* Pelagibacteraceae*, Raoultella, Rhizobium, Stenotrophomonas, Vibrionales*	Actinosynnemataceae*, Lentzea,* Nocardiaceae*, Nakamurella, Pseudonocardia*	*Akkermensia*	*Granulicella,* Acidobacteriaceae*, bacterium Ellin6075*	*Synergistia, Synergistales, Synergistes*
No	*Doreaformicigenerans, Faecalibacterium prausnitzii,* Lachnospiraceae*,* Lactobacillaceae*, Oscillospira Ruminococcus bromii,* Ruminococcaceae	Bacteroidaceae*, Balneolales* Rikenellaceae*,* Barnesiellaceae*,* Prevotellaceae Porphyromonadaceae	*Pseudomonadales, Raoultella*	—	—	—	—

The abundance of Proteobacteria was significantly higher in the irAEs group compared to the no-irAEs group. Additionally, Actinobacteria, Verrucomicrobia, Acidobacteria and Synergistetes phylum were only present in the fecal flora of patients with immune-related adverse events. This could be used as a potential marker to differentiate between irAEs and no-irAEs, as detailed in Table 4.

### 3.2 Clinical evidence linking bacterial biomarkers to different types of irAEs

To identify specific microbial biomarkers that can be used to classify patients with mild irAEs or severe irAEs, we found that the abundance of Firmicutes、Bacteroidetes, Actinobacteria and Proteobacteria were similar between the two groups, However, patients with severe irAEs had a visible abundance of Acidobacteria, while those with grades 1-2 irAEs had a higher abundance of Synergistetes at the phylum level, as detailed in Table 5. Therefore, these statistics suggested that patients with severe irAEs had an intestinal microbial community significantly different from those with mild irAEs.Wenhui Liu et al. the Bryan-Curtis intragroup distance of the no irAE group was smaller than both the mild irAEs and severe irAEs groups, however, there was no significant difference in α-diversity among them (Liu W. et al., 2021). These studies indice that patients without irAEs have a distinctly different gut microbial composition from those with mild and severe irAEs. And the compositions of microbiome could be used to be clinical tools to stratify patients during the treatment with checkpoint blockade therapy into groups with high and mild risk of irAEs. It is very valuable for surgeons to weigh the potential danger and advantages of immunotherapy. Further research is needed to explore and validate whether the regulation of the gut microbiome affects a variety of immune-mediated adverse events (Mao et al., 2021).

**TABLE 5:** Gut microbiome bacteria in patients with 1-2 irAEs and 3-4 irAEs, by phylum.

irae	Firmicutes	Bacteroidetes	Actinobacteria	Proteobacteria	Acidobacteria	Synergistetes
3-4	*Agathobacter, Succiniclasticum, Staphylococcus, Streptococcus, Thermoanaerobacteracea*	*Aquirestis, Dysgonomonas, Flavisolibacter,* Spirosomaceae	Actinosynnemataceae*, Lentzea, Nakamurella, Pseudonocardia*	*Acidicapsa,* Anaplasmataceae, *Chelativorans,* Coxiellaceae*,* Phyllobacteriaceae*,* Pelagibacteraceae*, Plesiomonas, Rhizobium, Stenotrophomonas, Vibrionales*	Acidobacteriaceae*, bacteriumEllin6075Granulicella*	—
1-2	*Anaerotruncus, Faecalibacterium,* Lactobacillaceae*, Phascolarctobacterium*	*Butyricimonas,* Odoribacteraceae*, Odoribacter*	Nocardiaceae	*Deltaproteobacteria* Pseudomonadaceae*, Raoultella*	—	*Synergistia, Synergistales, Synergistes*

## 4 Clinical application and potential challenges in modulating the gut microbiota

Faecal microbiota transplantation (FMT) is a process in which stools from healthy donors or previous stools from the same individual are transplanted into the gastrointestinal tract of recipients to balance or restore gut microbial composition (Tan et al., 2022).

### 4.1 FMT to boost the clinical efficacy of immunotherapy and mitigate immune-related adverse events

**FIGURE 1:**
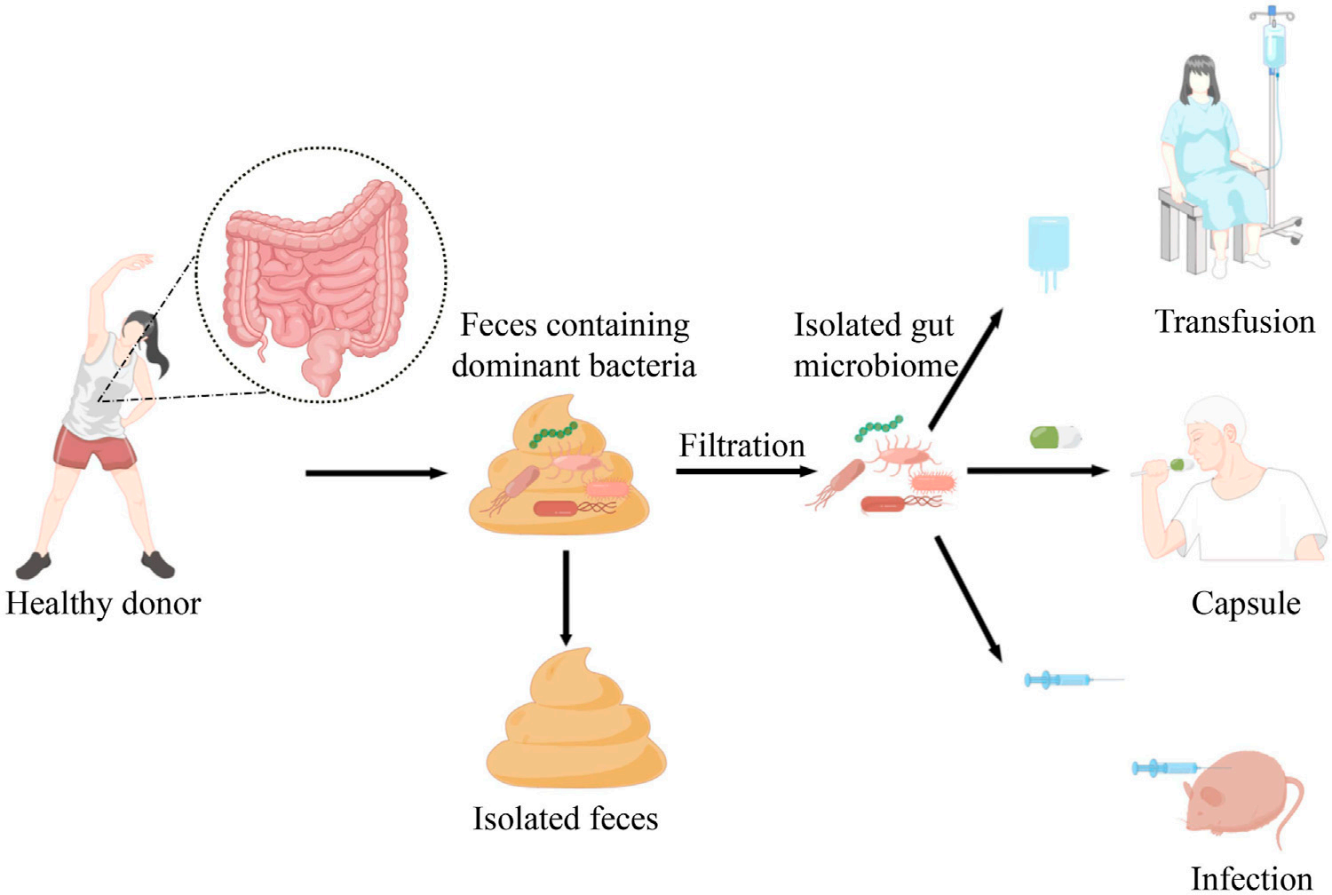
Faecal microbiota transplantation is a relatively mature approach isolating the dominant bacteria from healthy donor into the patients or mice with cancer. At present, there are three forms of faecal microbiota transplantation, including transfusion, oral administration or injection based on the manufactured bacterial fluids or capsules in order to reshape the vivo intestinal microecology.

In recent years, the emergence of resistance to immunotherapy and the occurrence of immune-related adverse events have posed great challenges for clinical immunotherapy. Several studies suggest that modulating intestinal microbiome can enhance immunotherapy response and reduce the occurrence of complications. Fecal microbiome transplantation is a relatively mature method to regulate microbiome and restore the richness of the recipient’s intestinal microbiome. Nowadays, there are three forms of faecal microbiota transplantation, including transfusion, oral administration or injection based on the capsules or manufactured bacterial fluids in order to reshape the vivo intestinal microecology, as shown in the Figure 1. Diwakar Davar et al. found that in FMT transplant-responding advanced melanoma patients circulating IL-8 downregulates. IL-8 is an immunosuppressive cytokine secreted by intratumoral and circulating myeloid cells, which correlates with poor prognosis with anti-PD1 use (Sanmamed et al., 2017; Schalper et al., 2020; Davar et al., 2021). Additionally, IL-8 was negatively correlated with increased levels of the beneficial bacteria *Faecalibacterium prausnitzii* and *A. muciniphila* in responders. Thus, FMT may adjust intestinal microecology and optimize immunotherapy, which can enhance the quality of life for patients with advanced malignant tumors and prolong their survival. Similarly, in an experiment with mice, mice that transplanted fecal microbiome from patients who had responded to anti-PD1 treatment were more active to immunotherapy and had a higher density of CD8^+^T cells after receiving treatment while those receiving stool from non-responsive patients developed resistance to ICIs (Gopalakrishnan et al., 2018b). According to the researches, FMT can provide a new therapeutic opportunity for patients with solid tumors who are resistant or less effective in immunotherapy. Moreover, FMT can be used to alleviate the irAEs during treatment. Yinghong Wang et al. reported the first successful ICI-associated colitis treatment case treated with fecal microbiome transplantation (FMT) in the University of Texas MD Anderson Cancer Center in 2018 (Wang Y. et al., 2018). With early insights into potential mechanisms, they revealed that FMT can be used to modulate the gut microbiome and improved symptoms of refractory ICI-associated colitis rapidly and significantly. Subsequently in 2020, National Comprehensive Cancer Network (NCCN) guidelines introduced FMT as an optional treatment for colitis refractory to immunosuppressant therapy based on institutional availability and expertise (Ianiro et al., 2020). Although early insights into the treatment of refractory colitis are provided, the study cohorts are very small and there are significant limitations. Given the widespread application of ICI across different cancer types, It is anticipated that there may be increasing incidence of ICI-associated colitis and other irAEs. Therefore, it is essential to carry out more investigations to assess the effectiveness of FMT and further mechanistic insight should be provided.

### 4.2 Limitations and risks of FMT

Although FMT has the advantage of increasing the chance of obtaining a long-term reset of the microbiome, it is important to note that there are some limitations and risks associated with the transfer of pathogenic microorganisms. In 2019, Zachariah DeFilipp et al. found that two patients who underwent FMT developed extended-spectrum beta-lactamase (ESBL)–producing *Escherichia coli* bacteremia, and one of them died soon (DeFilipp et al., 2019). Additionally, a systematic review reported five patients who developed infections after FMT (Shogbesan et al., 2018). Furthermore, the emergence of COVID-19 in the last 3 years has posed a challenge for fecal microbiome transplantation, as the virus has been detected in the stool of some asymptomatic infected individuals in a research (Nagy et al., 2020; Thompson et al., 2020).

The ineffectiveness of FMT may be due to several factors, such as a decrease in the patient’s immunity, the absence of taxa needed for therapy effectiveness in the FMT, and the disruption of the host microorganism due to graft failure (Davar et al., 2021). Therefore, it is essential to be aware of the potential risks and limitations of FMT.

### 4.3 Administration of FMT

The safety of FMT should be the primary consideration in clinical decision-making and more clinical studies should be carried to ensure the efficacy, particularly among immune-compromised patients. Additionally, the patient’s commensal background should be considered before receiving FMT, as primary intestinal mucosal commensal bacteria may interfere with the colonization of the complementary flora (Zmora et al., 2018). Furthermore, it is necessary to control the types and content of beneficial bacteria used for FMT materials and the management of probiotics to produce standardized specimens and minimize potential contamination (Pierrard and Seront, 2019). Last but not to be neglected is that considering the heterogeneity of the relevant studies, a large number of trials are needed to explore the clinical implications of FMT (Pierrard and Seront, 2019).

### 4.4 Other strategies to modulate the gut microbiota in patients with cancer and treated with the ICIs

Similarly, the use of antibiotics could alter gut microbiota diversity and composition leading to dysbiosis, which may affect effectiveness of ICI. For example, patients with renal cell carcinoma (RCC), non-small-cell lung cancer (NSCLC), hepatocellular carcinoma (HCC) and so on often obtained lower OS and PFS if they were given antibiotics prior to anti-programmed cell death ligand-1 mAb monotherapy or combination therapy (Derosa et al., 2018; Schett et al., 2020; Ochi et al., 2021). Those reveal the strong relationship between the broad-spectrum ATB class and poor efficiency. Still, considering the homogeneous populations, more researches shall be carried in order to clarify these issues. Meanwhile, Clinicians shall carefully consider the use of antibiotics in cancer patients treated with ICIs (Crespin et al., 2023).

Nowadays, the use of probiotics, prebiotics and synbiotics largely enriches the interventional approaches to manipulate the microbiota. It is well known that probiotics are defined as live microorganisms which when administered in adequate amounts confer a health benefit on the host. *Lactobacillus* and *Bifidobacterium* are the most commonly probiotics. *Lactobacillus delbrueckii* can induce cell apoptosis and inhibit the growth of human colon cancer cell (Wan et al., 2014). *Lactobacillus* spp. in colorectal cancer modulate host immunity, inhibit cell proliferation to realize anti-cancer (Wong and Yu, 2019). However, it is well known that not all *Lactobacilli* are probiotics because probiotic effects are strain-dependent. *Bifidobacterium* was demonstrated as an unexpected role for enhancing anti-tumor immunity in studies of Ayelet Sivan et al. (Sivan et al., 2015), which can improve the response of PD-1/PDL-1 inhibitors (Zhuo et al., 2019). Therefore, the advantages of probiotics are unprecedented. However, the health value of probiotics should be assessed combining multiple factors, such as clinical parameters, baseline commensal background and microbiome features considering the resistance to probiotics colonization (Zmora et al., 2018; Langella and Chatel, 2019).

Another way to enrich gut microbes that promote anti-tumor and bring benefits for consumers is through prebiotics. To date all reported prebiotics are carbohydrates. The quintessential prebiotics are inulin-type fructans, fructo-oligosaccharides (FOS) and galacto-oligosaccharides (GOS) (Huxley et al., 2009). They can be obtained from certain grains, fruits, nuts and vegetables, which can promote substantial alterations in the composition of fecal microbiota and commensal bacteria to produce relative metabolites (Derosa et al., 2021; Tan et al., 2022).

Synbiotics are a combination of prebiotics and probiotics that are believed to have a synergistic effect by inhibiting the growth of pathogenic bacteria and enhancing the growth of beneficial organisms. Rafter J. et al. have discovered that the combination of prebiotic inulin and the probiotics *Lactobacillus* rhamnosus GG and Bifidobacterium lactis Bb12 may change the composition of gut microbiota in patients with colonic polyps, which improved epithelial barrier function (Wong and Yu, 2019).

Efforts are required to further understand the mechanisms between the composition of intestinal microbiota and the efficacy of immunotherapy. Future research shall shed light on different animal models and prospective clinical studies to help further understand the role of intestinal microbiota. The composition of intestinal microbiota may be an essential component for cancer therapy in this fast-moving era.

## 5 Conclusion

Numerous studies have confirmed that gut flora plays a crucial role in the immunotherapy of cancer. Identification of specific dominant and ineffective flora can be an important basis for judging tumor prognosis and adverse events; Additionally, beneficial fecal microbiome transplantation both moderates gut flora and significantly improves the outcome. However, it is a promising therapeutic approach that still requires a very cautious and low-key approach due to the different functions of the gut microbiota in the body as a whole, and needs to be combined with clinical studies to assess the relative contribution of pre-existing bacteria that may promote transplantation versus those that against transplantation as well as the need to standardize sample configuration procedures (Lam and Goldszmid, 2021).

Targeted at immunotherapy, it is of great necessity to clarify the specific bacteria that influence the effect of immunotherapy and consider the dynamic nature of microbial communities to determine the optimal sampling point for predicting efficacy and toxicity, as well as the need to standardize sampling procedures. Furthermore, it is essential to establish a unified standard for sequencing and bioinformatics analysis to screen out prognostic biomarkers with high sensitivity and specificity.

In addition, future studies are needed to explore how basic research can be effectively translated into clinical applications, whether gut flora can be used as a potential marker for cancer, the mechanism of patient response differences for the same class of bacteria shown in different studies and how to intervene in the gut flora to overcome the challenge of patient drug resistance and so on, which may maximize immunotherapy and further reduce the incidence of immune related adverse events.
